# Traditional Chinese medicine treatment in sinusitis after radiotherapy for nasopharyngeal carcinoma: a systematic review and network meta-analysis

**DOI:** 10.1007/s00405-023-08221-4

**Published:** 2023-09-14

**Authors:** Yihan Chen, Jianfeng Liu

**Affiliations:** 1 China-Japan Friendship Hospital, Beijing University of Chinese Medicine, Beijing, China; 2 Department of Otolaryngology-Head and Neck Surgery, China-Japan Friendship Hospital, Beijing, China

**Keywords:** Nasopharyngeal carcinoma, Network meta-analysis, Radiation sinusitis, Systematic review, Traditional Chinese medicine

## Abstract

**Background:**

Radiation sinusitis after radiotherapy for nasopharyngeal carcinoma occupies a large proportion and affects the subsequent therapeutic process as well as diagnosis, which can be improved by traditional Chinese medicine treatments.

**Methods:**

Based on the relevant clinical randomized controlled trials (RCTs) from eight databases, a network meta-analysis (NMA) in a frequentist framework was constructed after study selection, data extraction, and quality evaluation of the included studies. The outcomes included total effect, the Lund Kennedy score of nasal endoscopy and the Lund Mackay score of sinus CT.

**Results:**

For total effect, the order of probability for the effect is: external herbal medicine + herbal medicine orally > nasal saline + herbal medicine orally > herbal medicine orally > external herbal medicine > external herbal medicine + nasal saline > nasal saline + western medicine orally > none > nasal saline. For the Lund Kennedy score, the order of probability for the effect is: nasal saline + herbal medicine orally > herbal medicine orally > external herbal medicine > none > nasal saline. For Lund Mackay score, the order of probability for the effect is: herbal medicine orally > nasal saline + western medicine orally > nasal saline + herbal medicine orally > nasal saline > external herbal medicine.

**Conclusions:**

Herbal medicine taken orally and through nasal cavities combined with nasal saline has a better clinical effect than a single intervention for total effect and Lund Kennedy score. As the classification of this research is relatively macro and the sample size is insufficient, further higher-quality studies are needed to verify the conclusion.

**Trial registration:**

PROSPERO ID: CRD42022384113, 2022-12-25.

**Supplementary Information:**

The online version contains supplementary material available at 10.1007/s00405-023-08221-4.

## Background

Nasopharyngeal carcinoma (NPC) is a highly prevalent malignant tumor of the head and neck in China, with an incidence rate of approximately 2–3 times higher for men than for women and significant geographical differences, most (98%) of which are poorly differentiated squamous carcinomas [1]. Due to the anatomical features of the nasopharynx and sensitivity to radiation, according to the guidelines of the Chinese Society of Clinical Oncology (CSCO) and National Comprehensive Cancer Network (NCCN), early and locally advanced NPC prefer radiotherapy. Common complications after radiotherapy for NPC include acute radiation mucositis, radiation dermatitis, radiation salivary gland injury, acute ear injury and radiation brain necrosis [2]. Currently, intensity-modulated radiotherapy (IMRT) has become the main radiotherapy treatment for NPC to improve local control and overall survival and to reduce damage to surrounding normal tissues [3]. Due to the large area irradiated and the total dose for the hidden location of the nasopharynx as well as concomitant lymph node metastases, most (55.9%) 6-month post-therapeutic patients suffer from nasal congestion, runny nose, loss of smell, dizziness and headache, and foul-smelling oral cavity and nose because of radiation sinusitis. The incidence rises to approximately 70% 1–3 years after radiotherapy, which severely affects quality of life and rarely heals [4]. Early diagnosis of NPC recurrence is also extremely difficult due to the large amount of secretions and necrotic crusted tissue in the nasal cavity and nasopharynx [5]. Research found that radiation sinusitis was an important predictor of disease-free survival, local control rate, distant metastasis control and high negative predictive value (97.5%) [6] for local recurrence, so preventing or mitigating radiation sinusitis is a significant part of the treatment process for NPC.

Existing studies suggest that the main mechanisms of sinusitis secondary to radiotherapy for NPC are dryness or congestion of the nasal mucosa, impaired ciliary function, nasal ventilation and sinus drainage, and decreased immune function due to malignancy and radiotherapy. Common treatments include nasal irrigation, oral vasoconstrictors or antibacterial drugs, and nasal endoscopic surgery [7], but there is still much room and urgent research to be done due to irreversible damage to nasal mucosa and reduced ciliary transmission. The current evidence on traditional Chinese medicine (TCM) for radiation sinusitis after radiotherapy for NPC is not comprehensive. Utilizing network meta-analysis and existing randomized controlled trials (RCTs), this paper conducts a comparative analysis of different interventions of TCM for radiation sinusitis after radiotherapy for NPC (external herbal medicine, herbal medicine orally, external herbal medicine + nasal saline, external herbal medicine + herbal medicine orally, nasal saline + herbal medicine orally) and clinical conventional interventions (nasal saline, none, nasal western medicine/orally). A comprehensive efficacy comparison and analytical ranking were performed to provide visual data for the clinical treatment of sinusitis after radiotherapy for NPC.

This study has been registered in PROSPERO, a prospective systematic evaluation registry created by the National Institute for Health Research Centre for Evaluation and Dissemination (CRD). Registration Number: CRD42022384113 (https://www.crd.york.ac.uk/prospero/display_record.php? RecordID = 384,113).

## Methods

### Search strategy

Researchers searched CNKI, WanFang Data, CQVIP databases, Sinomed, PubMed, Embase and Cochrane Library until December 14, 2022. The references of the included studies were traced to obtain other related studies to supplement the included studies. The search was carried out by combining subject terms and free words. All RCTs of TCM in the treatment of radiation sinusitis after radiotherapy for NPC were collected. The search string was built as follows: (Nasopharyngeal Carcinoma OR Nose Neoplasms) AND (Radiotherapy OR Radiosurgery) AND (Sinusitis OR Paranasal Sinus Diseases) AND (Traditional Chinese Medicine OR Herbal Medicine OR Acupuncture). Taking PubMed as an example, the retrieval strategy is shown in Supplemental Table 1.

### Inclusion and exclusion criteria

Inclusion and exclusion criteria were formulated based on the PICOS principle (P-Population/Participant; I-Intervention; C-Comparison; O-Outcome; S-Study design).

Inclusion criteria: ① Study design: published RCTs. There was just one option for Chinese or English. ② Population/participant: nasopharyngeal cancer patients treated with radiation. The diagnostic criteria referring to nasopharyngeal carcinoma in Diagnostic and Treatment Standards for Common Malignancies in China and nasal abyss in Diagnostic Efficacy Criteria for TCM. Or in line with newly emerging radiation sinusitis (within 1–2 years from the end of radiotherapy), possessing nasal symptoms (nasal congestion, runny nose or postnasal drip, facial pain or pressure, headache, loss of smell or sense of smell, blood in the nose or mouth, and nasal odor) and definitely diagnosed by auxiliary examinations (e.g., anterior rhinoscopy, endoscopy, imaging, biopsy and bacteriological examination) [8]. Patients’ gender, age, nationality, ethnicity, occupation, level of education, illness course were not subject to restrictions. Participants in the identical RCT were permitted to have hypertension, diabetes, hyperlipidemia and other underlying conditions, and the baseline of studies was balanced (*P* > 0.05). ③ Intervention and comparison: the primary intervention was traditional Chinese medicine (acupuncture, herbal medications administered orally or by nasal irrigation, etc.). Saline nasal irrigation, macrolide antibiotics and similar standard medical care are comparators (without therapy or a blank control). ④ Outcome: the total effect is the main outcome. The total effect is equal to (the number of “cured” and “improved”/the total sample size)*100%. Additional outcomes are nasal endoscopy (Lund Kennedy score) and sinus CT (Lund Mackay score).

Exclusion criteria: ① The patient’s tumor lesions were illegible or merged with tumor lesions from other places, blood system illnesses, immune system illnesses, and with serious dysfunction of the heart, liver, lung, kidney, and hematological system. ② The experimental groups or controlled groups were applied other therapies besides herbal remedies, western medicine and acupuncture, such as psychotherapy. ③ Continuous publishing. ④ Studies lacking comparable data or that are not accessible to researchers. ⑤ The incidence of loss to follow-up or drop-off was more than 50%, the data of outcome was evidently missing or incorrect or the efficacy evaluation was unclear.

### Study selection and data extraction

Trained researchers were separated into groups to screen studies, extract data and cross-check the data independently. Discussions were held to settle any disputes. Subsequently, data were extracted into a unified spreadsheet, and the extraction contents contained the following: ① Basic information about the included studies: title, first author’s name, publication year, Study ID, data source, etc.; ② Baseline characteristics of the subjects: age, stage of disease, sample size of each group, etc.; ③ intervention: treatment (including TCM either in conjunction with or without Western medicine), modality (including orally, nasal rinse, nebulized inhalation, etc.), medicine composition, etc.; ④ relative information about risk assessment of bias: random method, diagnostic and efficacy evaluation criteria, drop-off and follow-up scenarios, etc.; ⑤ outcome: total effect, nasal endoscopy (Lund Kennedy score) and sinus CT (Lund Mackay score).

### Risk of bias

Researchers evaluated the quality of the included studies according to the bias risk assessment tool, namely, ROB2, which was advised by Cochrane Handbook 5.1.0. The evaluation concerned five main areas: ① the randomization process; ② deviations from intended interventions; ③ missing outcome data; ④ measurement of the outcome; ⑤ selection of the reported results. The answers to questions involved with the five domains were provided as Yes (Y), Probably Yes (PY), Probably No (PN), No (N), and No Information (NI).

### Statistical analysis

Researchers utilized Stata/MP 17.0 software to construct NMA in a frequentist framework. For dichotomous variables (total effect), the odds ratio (OR) was chosen as the effect size. For continuous variables (Lund Kennedy score of nasal endoscope, Lund Mackay score of sinus CT), the mean difference (MD) was chosen as the effect size. Meta-analysis was carried out by calculating the effect values and their 95% credibility interval (CI).

The network map was created to depict the comparator arm and relationship of various interventions, with “points” and “lines” weighted according to overall sample size and number of studies that directly compared the connected two interventions.

An *I*^2^ (I-squared) test was used to measure heterogeneity. According to Higgins et al. [9], the likelihood of heterogeneity between 0 and 100% increased as *I*^2^ increased. There was mild heterogeneity when *I*^2^ = 25%, moderate heterogeneity when *I*^2^ = 50%, and a high degree of heterogeneity when *I*^2^ = 75%. The Cochrane Handbook deemed that when *I*^2^ > 50%, the research was considered to be heterogeneous, and a random effects model was applied. When *I*^2^ < 50%, the fixed effects model was applied. If the heterogeneity was high, further subgroup analysis and meta-regression were performed to examine the sources of heterogeneity.

Each closed loop in the network map underwent an inconsistency test. The inconsistency factors (IFs) and 95 percent credibility interval (CI) and the heterogeneity parameter *t*^2^ (*t* = SD) of each loop were calculated to analyze whether there was inconsistency in each closed loop. The closer the IF gets to 1, the more consistent the results between different studies. If the lower limit of the 95% CI was 1, the direct comparison results were consistent with the indirect comparison results [10].

Set “None” or “Nasal saline” as the initial control intervention. The direct and indirect comparative findings of several interventions were displayed using an interval prediction graph and an inverted triangle diagram. The area under the curve, which is referred to the graph known as SUCRA, was associated with treatment ranking (surface under the cumulative ranking curve). The intervention will have a better impact on a larger area.

## Results

### Results of the search process

**Fig. 1:**
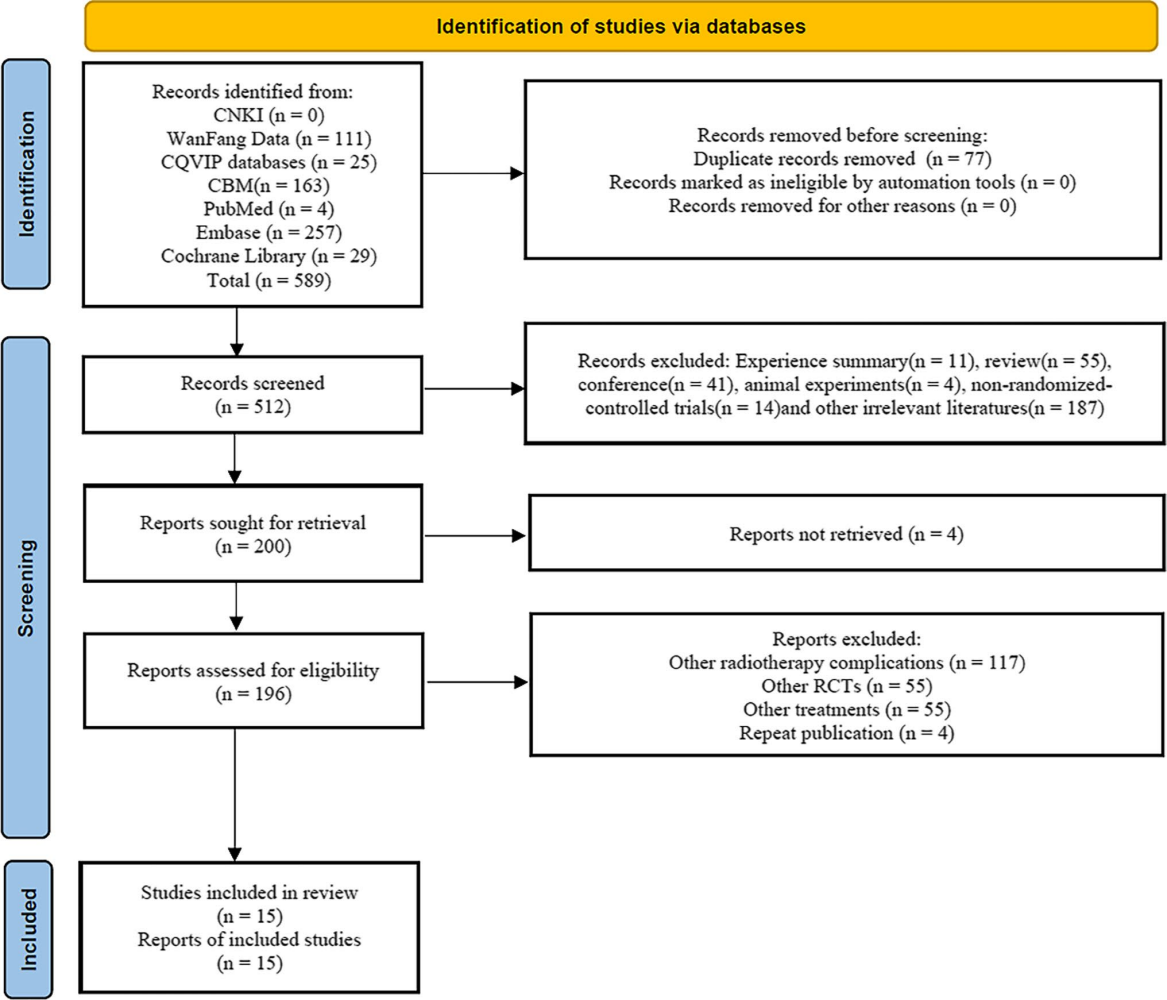
Search process depicted by the PRISMA flowchart

The total amount of obtained records was 589, which were imported into NoteExpress 3.2.0, and 512 records were obtained after duplicates were removed. Researchers simply screened titles and abstracts and then obtained 200 records after excluding experience summaries, reviews, conferences, animal experiments, nonrandomized controlled trials and other irrelevant literature. The remaining full texts were further screened, and 15 records were left after excluding those that deviated from required radiotherapy complications, outcomes or interventions, as well as repeated publications and unavailable ones. 15 RCTs were ultimately included in the meta-analysis, and the process is depicted in Fig. 1.

### Characteristics of the included studies

15 articles were included in the research with a total of radiation sinusitis patients, 656 for experimental groups and 654 for comparator groups. The total effect was the main outcome, while the Lund Kennedy score and Lund Mackay score were the secondary. 13 studies reported the total effect, 6 studies reported the Lund Kennedy score, and 5 studies reported the Lund Mackay score. As demonstrated in Supplemental Table 2, two studies [11, 12] reported adverse effects, and only one study [13] reported follow-up results.

Eight kinds of interventions are involved: A–external herbal medicine (including nasal rinse, topical application, ultrasonic nasal inhalation and sinus negative pressure replacement), B–Nasal saline (including nasal rinse, ultrasonic nasal inhalation and sinus negative pressure replacement), C–None, D–Herbal medicine orally, G–External herbal medicine + Nasal saline, H–External herbal medicine + Herbal medicine orally, I–Nasal saline + Herbal medicine orally, J–Nasal saline + Western medicine orally. Western medicine mainly consisted of Budesonide + Mucosolvan through atomized inhalation and Roxithromycin taken orally. Intervention of nasal saline included saline or vitamin B12 added. Topical application of herbal medicine on skin applied Golden Yellow Powder. Contents of herbal medicine through nasal cavities included Radix Gentianae, *Hedyotis diffusa*, Bletillae Rhizoma, Radix Angelicae Dahuricae, Herba Menthae, Radix Glehniae, Fructus Xanthii, Radix Bupleuri, Paeoniae Radix Rubra, Rhizoma ChuanXiong, Angelicae Sinensis Radix, Vespae Nidus, Poria, Radix Rhizoma Glycyrrhizae, Testudinis Carapacis Et Plastri, Ecliptae Herba, Radix Astragali, Radix Scutellariae, Lonicerae Japonicae Flos, Schizonepetae Herba, Platycodonis Radix, Gentianae Radix Et Rhizom, Resina Draconis, Ephedrae Herba, Radix Ophiopogonis, Akebiae Caulis, Mori Cortex, Acori Tatarinowii Rhizoma, Persicae Semen, Radix Trichosanthis, Smilacis Glabrae Rhizoma, Flos Magnoliae, Flos Chrysanthemi Indici, Houttuynia cordata Thunb, Spina Gleditsiae, Fructus Gardeniae. Patent herbal medicine through nasal cavities included Erhuang Decoction, Shanbangtong Liquid, Zao-Jiao-Ci Granule Solution. Contents of herbal medicine taken orally included Radix Angelicae Dahuricae, Herba Menthae, Radix Glehniae, Fructus Xanthii, Rhizoma ChuanXiong, Radix Rhizoma Glycyrrhizae, Radix Puerariae, Radix Astragali, Radix Scutellariae, Chrysanthemi Flos, Mori Folium, Flos Magnoliae. Patent herbal medicine taken orally included Biyan Qingdu Granules, Biyuanshu Oral Liquid; nasosinusitis relieving oral liquid, Xanthium Powder, Shashen Maidong Decoction, Xiangsha Liujunzi Decoction combined with Ganlu Xiaodu Drink.

### Risk of bias and certainty of evidence

**Fig. 2:**
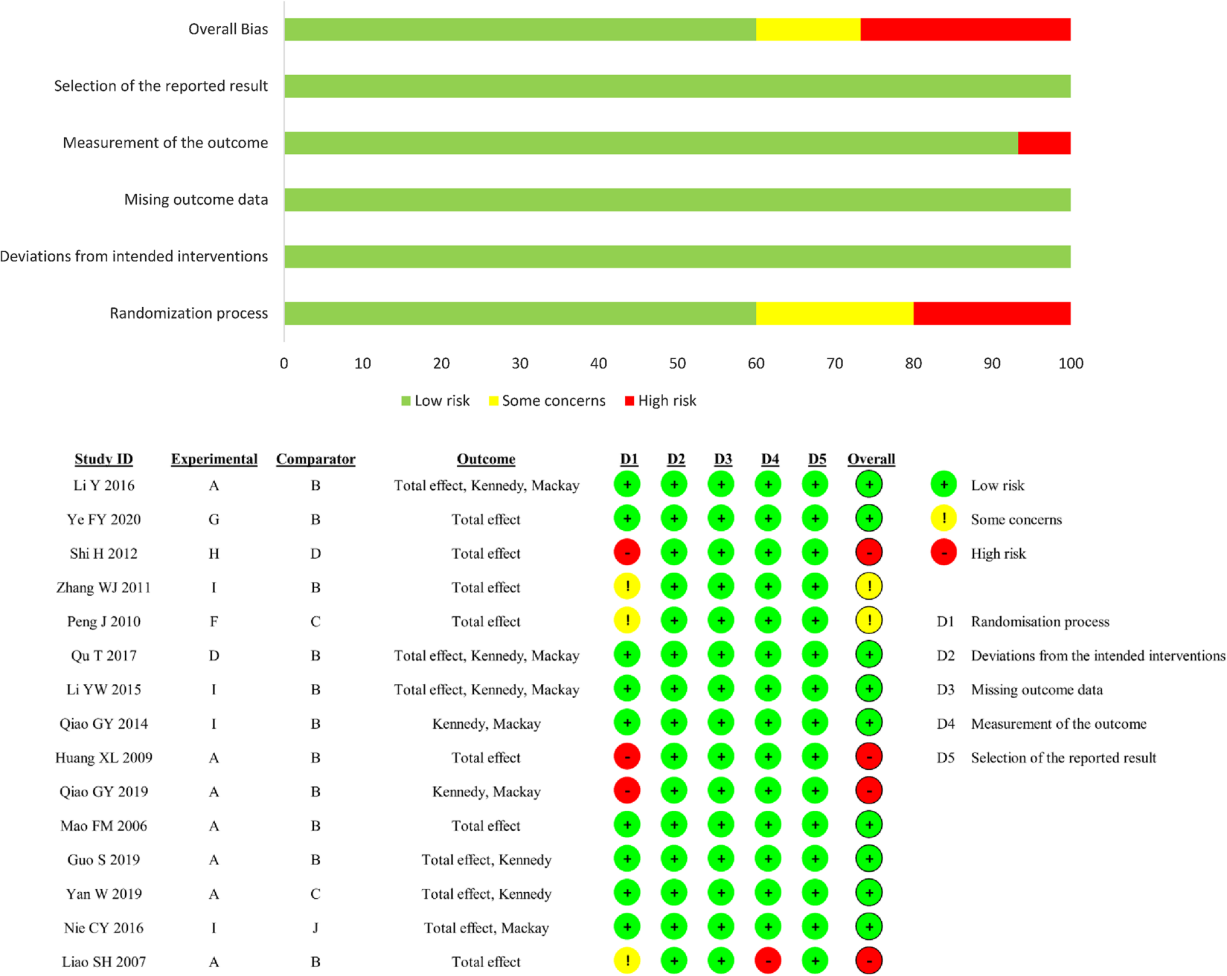
Risk of bias assessment results of the included studies (A-external herbal medicine, B-nasal saline, C-none, D-herbal medicine orally, G-external herbal medicine + nasal saline, H-external herbal medicine + herbal medicine orally, I-nasal saline + Herbal medicine orally, J-nasal saline + western medicine orally) Over 60% of the included studies were evaluated as low risk overall.

Researchers used the bias risk assessment tool, named ROB 2, recommended by Cochrane 5.1.0. A total of five aspects of the original study were assessed, including the randomization process, deviation from intended interventions, missing outcome data, measurement of the outcome, and selection of the reported result. Included studies were classified as high quality, low quality or unknown risk bias. There were four studies rated as high quality (Fig. 2), one [14] for possible inconsistencies at baseline and subjective outcome indicators according to clinician judgement, and three [13, 15, 16] for participants grouped according to the time or order of attendance. There were two studies rated as having an unknown risk of bias, one [17] with an unclear grouping approach and another [18] with possible inconsistencies at baseline.

### Total effect

#### Network structure

**Fig. 3:**
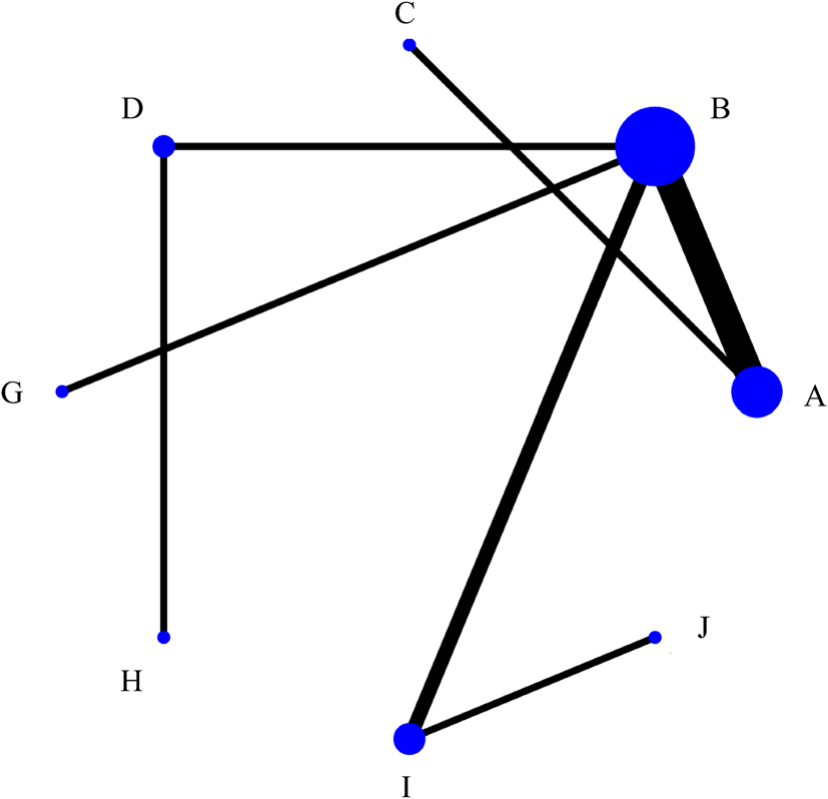
Network diagram comparing treatment outcomes for total effect (A-external herbal medicine, B-nasal saline, C-none, D-herbal medicine orally, G-external herbal medicine + nasal saline, H-external herbal medicine + herbal medicine orally, I-nasal saline + herbal medicine orally, J-nasal saline + western medicine orally) The diameter of each dot represents the proportional total weight of all trials in the network that investigated that intervention, while the thickness of each line connecting two interventions is proportional to the number of trials that investigated that pair of interventions

A total of 13 studies reported the total effect (two forward-looking studies were not included in the statistical analysis), with a total of 22 arms involving 984 patients. Figure 3 depicts the comparative relationship between different interventions. The dots represent the total number of samples in all studies using this treatment. The lines represent the amount of research evidence that directly compared the two treatments connected. Indirect comparative analysis was carried out based on network structure for two unconnected interventions. Studies included eight kinds of interventions: A-external herbal medicine, B-nasal saline, C-none, D-herbal medicine orally, G-external herbal medicine + nasal saline, H-external herbal medicine + herbal medicine orally, I-nasal saline + herbal medicine orally, J-nasal saline + western medicine orally.

#### Contribution plot

Supplemental Fig. 1 displays the contribution of each direct comparison result to the comprehensive comparison result of NMA based on the total effect. For example, 50.0 means the contribution rate of the direct comparison between intervention A (external herbal medicine) and intervention B (nasal saline) for comparing the efficacy of intervention A (external herbal medicine) and intervention D (herbal medicine orally) is 50.0%.

#### Testing for heterogeneity and inconsistency

According to the results of the heterogeneity test, *I*^2^ = 24% < 25%, *P* = 0.0233 < 0.05, regarded as low heterogeneity, NMA was carried out under the fixed effects model. A consistency model was selected for the NMA of the total effect because no closed loop was formed.

#### Network meta-analysis

**Fig. 4:**
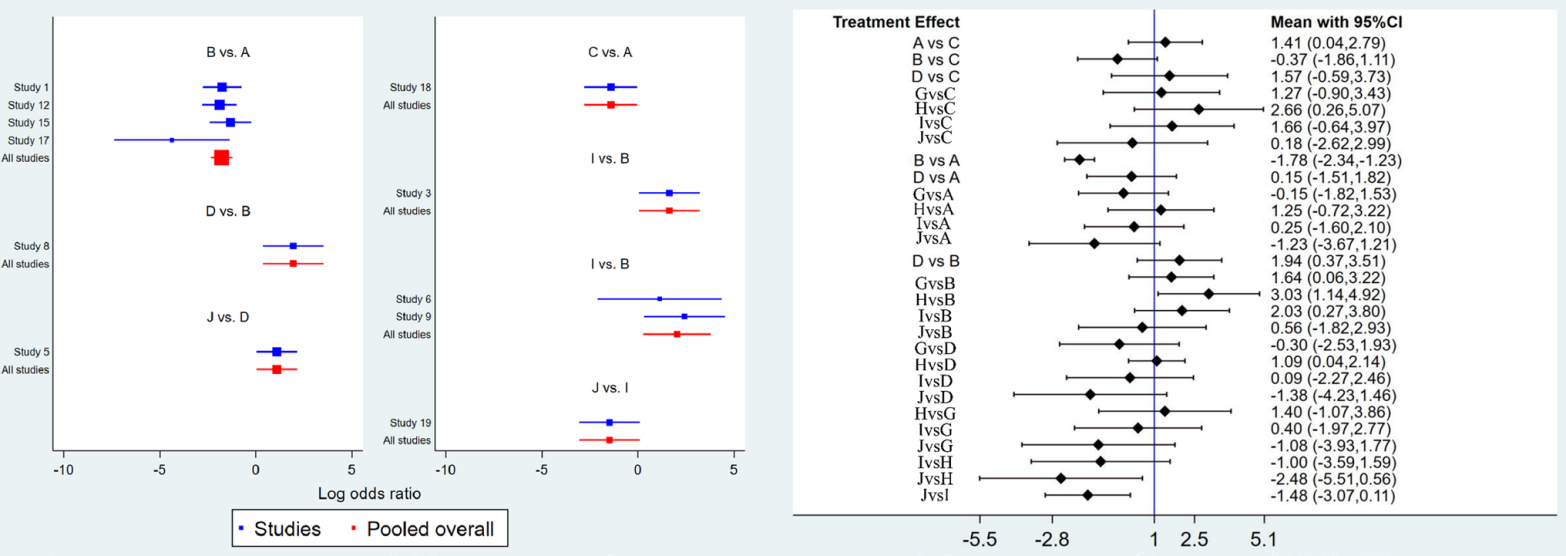
Forest plot of treatment differences for total effect (A-external herbal medicine, B-nasal saline, C-none, D-herbal medicine orally, G-external herbal medicine + nasal saline, H-external herbal medicine + herbal medicine orally, I-nasal saline + herbal medicine orally, J-nasal saline + western medicine orally) The ineffectiveness line (vertical line, *X* = 1) indicates an equal ratio. Each horizontal line connects the upper and lower limits of the 95% confidence interval for the study, and the length of the lines indicates the range of the confidence interval. If the line crossed = 1, the study was not statistically significant. If the line totally falls on the left side of *X* = 1, it indicates worse efficacy, and the right side indicates the opposite. The diamond-shaped blocks are locations corresponding to the OR values

**Fig. 5:**
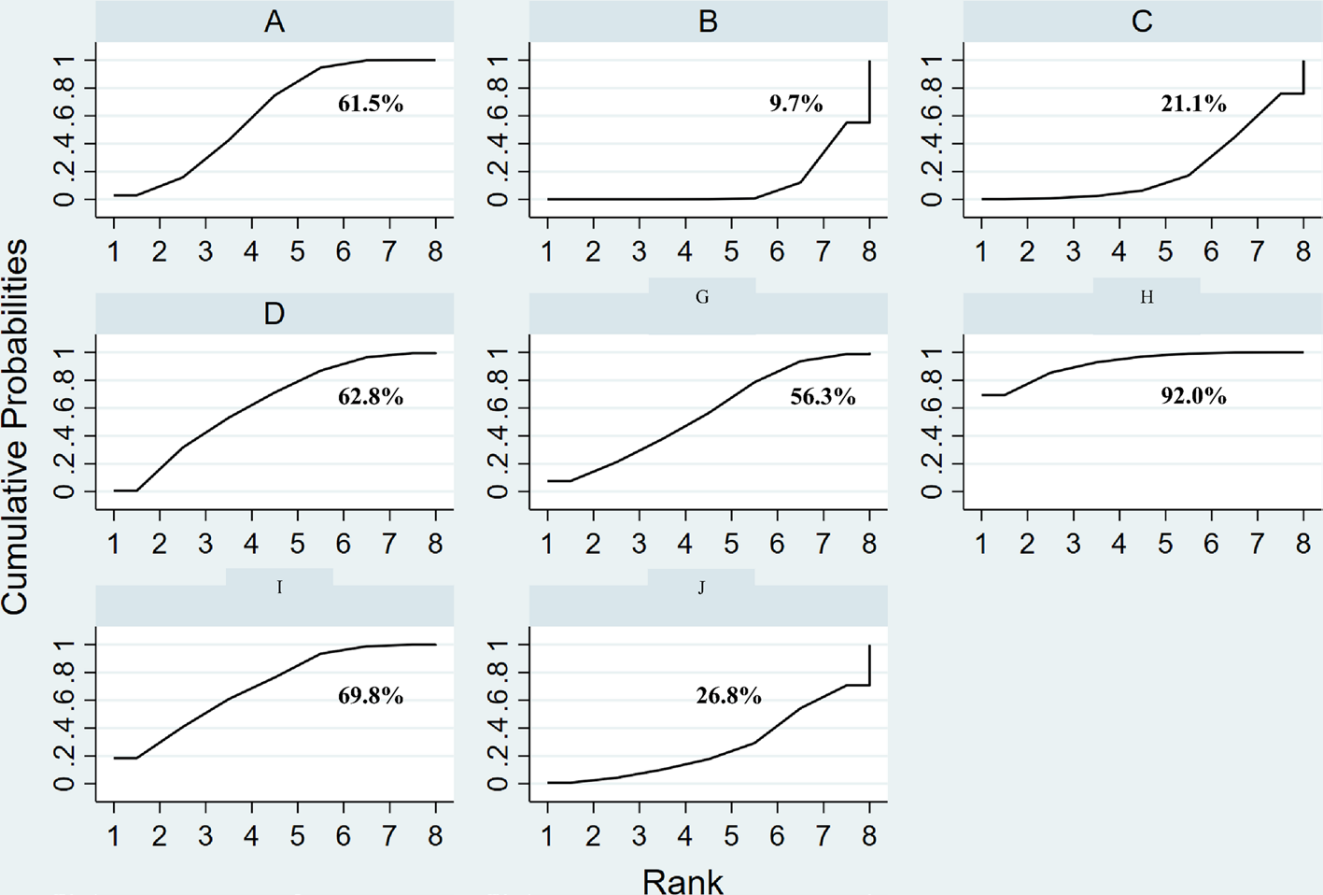
Surface under the cumulative ranking curves for the total effect (A-external herbal medicine, B-nasal saline, C-none, D-herbal medicine orally, G-External herbal medicine + nasal saline, H-external herbal medicine + herbal medicine orally, I-nasal saline + herbal medicine orally, J-nasal saline + western medicine orally) The *Y* axis represents cumulative probability, and the *X* axis represents rank. When comparing the cumulative probability of the same control rank, the higher ranking (5 → 1) with a higher cumulative probability means a better curative effect

Figure 4 (left) displays different combined studies and combined effect size. The results shown in Fig. 4 (right) indicate that the curative effects of external herbal medicine and external herbal medicine + herbal medicine orally are better than those of nasal saline, external herbal medicine + herbal medicine orally and nasal saline + herbal medicine orally are better than those of nasal saline + western medicine orally. The differences in the remaining comparisons are not statistically significant. The results mentioned above can also be obtained from the inverted triangle diagram (Table 1). The surface under the cumulative ranking curve (SUCRA) (Fig. 5) depicts external herbal medicine + herbal medicine orally is most likely to be the most effective intervention for treatment

**Table 1:**
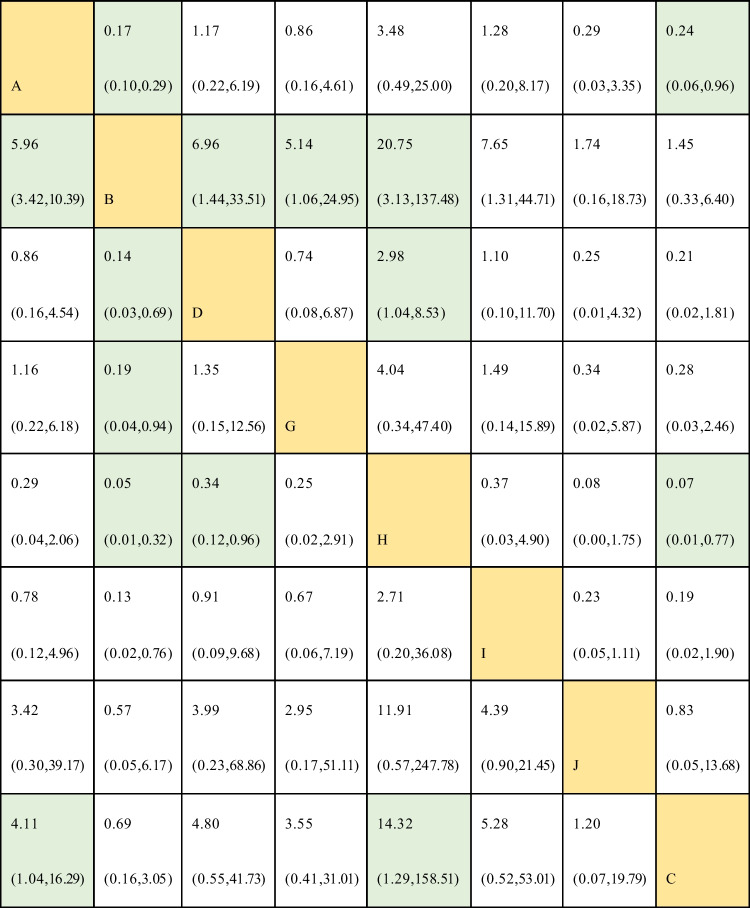
Inverted triangle diagram for total effect

The yellow table cells represent interventions. (A-external herbal medicine, B-nasal saline, C-none, D-herbal medicine orally, G-external herbal medicine + nasal saline, H-external herbal medicine + herbal medicine orally, I-nasal saline + herbal medicine orally, J-nasal saline + western medicine orally). The green table cells represent the combined effect size, which can be referenced to compare the curative effect between the interventions in the column and the line

#### Small sample effect and bias

The comparison-correction funnel plot (Supplemental Fig. 2) shows that most dots are distributed on both sides of the vertical line of *X* = 0 at the top of the funnel diagram and concentrated in the middle. Two studies (A-external herbal medicine compared with B-nasal saline, B-nasal saline compared with D-herbal medicine orally) were distributed at the bottom of the funnel diagram away from *X* = 0, which means a possible small sample effect and publication bias.

### Lund Kennedy score

#### Network structure

A total of six studies reporting the Lund Kennedy score of nasal endoscopy were included, with a total of 12 arms involving 452 patients. Supplemental Fig. 3 (left) shows the comparative relationship between the different interventions. The studies included six interventions: external herbal medicine, nasal saline, none, herbal medicine orally, nasal saline + herbal medicine orally, and nasal saline + Western medicine orally.

#### Contribution plot

Supplemental Fig. 4 (left) depicts the contribution of each direct comparison result to the comprehensive result of the NMA based on the Lund Kennedy score.

#### Testing for heterogeneity and inconsistency

According to the heterogeneity results, *I*^2^ = 97.68% > 75%, *P* = 0.3654 > 0.05, regarded as heterogeneity, and NMA was carried out under the randomized effects model. Due to the small sample size of the included studies applying the Lund Kennedy score, subgroup analysis could not be performed. A consistency model was selected for the NMA of the Lund Kennedy score because no closed loop was formed.

#### Network meta-analysis

Supplemental Fig. 5 (left) depicts the results of direct and indirect comparisons, which shows no statistical significance. The curative effects of nasal saline + Herbal medicine orally were better than those of nasal saline, as shown in the inverted triangle diagram (Supplemental Table 3). The SUCRA in Supplemental Fig. 6 (left) shows that nasal saline + Herbal medicine orally is most likely to be the most effective intervention.

#### Small sample effect and bias

The comparison-correction funnel plot in Supplemental Fig. 7 (left) shows that most dots are symmetrically distributed on both sides of the vertical line of *X* = 0, while the bottom of the funnel diagram indicates the possibility of a small sample effect.

### Lund Mackay score

#### Network structure

A total of five studies reporting the Lund Mackay score of sinus CT were included, with a total of 10 arms involving 482 patients. Supplemental Fig. 3 (right) shows the comparative relationship between the different interventions. Studies included five interventions: external herbal medicine, nasal saline, herbal medicine orally, nasal saline + herbal medicine orally, and nasal saline + western medicine orally.

#### Contribution plot

Supplemental Fig. 4 (right) depicts the contribution of each direct comparison result to the comprehensive result of NMA based on the Lund Mackay score.

#### Testing for heterogeneity and inconsistency

Due to the small sample size of the included studies applying the Lund Mackay score, insufficient material was shown during heterogeneity analysis, and NMA was carried out under the fixed effects model. A consistency model was selected for the NMA of the Lund Mackay score because no closed loop was formed.

#### Network meta-analysis

Supplemental Fig. 5 (right) and the inverted triangle diagram (Supplemental Table 4) depict the results of direct and indirect comparisons, which show no statistical significance. The SUCRA in Supplemental Fig. 6 (right) shows that Herbal medicine orally is most likely to be the most effective intervention.

#### Small sample effect and bias

The comparison-correction funnel plot in Supplemental Fig. 7 (right) displays that most dots are symmetrically distributed on the vertical line of *X* = 0, and the dot of one study (nasal saline compared with nasal saline + herbal medicine orally) is at the bottom of the funnel diagram, indicating the possibility of a small sample effect and publication bias.

## Discussion

### Implications of the findings

NPC is the most frequent head and neck cancer in China and radiation therapy is the predominant clinical approach. After irradiation, the majority of patients develop nasal symptoms due to radiation sinusitis after radiotherapy, which lowers the quality of life and makes it more challenging to diagnose the recurrence of NPC and later recovery. Radiation therapy can weaken nasal transport and defense function by damaging normal nasal mucosal tissues and induce acute radiation reactions by thinning mucosal vascular walls, increasing permeability, edema and bleeding, which are followed by radiation sinusitis [19].

Traditional Chinese medicine classifies radiation sinusitis as “nasal abyss”. TCM theory believes that radiation is an external evil of heat and toxicity. The evil will be stagnant in the sinuses as heat and toxicity damage physiological fluids and Qi, causing a yin deficiency of the lung and stomach. Meanwhile, the spleen loses the ability to transport and transform water and fluid, and dampness and heat steaming upwards accumulate in the sinuses, which results in radiation sinusitis. TCM therapy can improve symptoms of radiation sinusitis and body immune function and reduce recurrence and metastasis of nasopharyngeal carcinoma with few side effects. In recent years, TCM combined with western medicine in the treatment of sinusitis after radiotherapy for NPC has been gradually confirmed and adopted by researchers and clinicians.

We searched relevant articles and utilized NMA to discuss and rank the efficacy of different interventions of TCM combined with western medicine in the treatment of radiation sinusitis after radiotherapy for NPC in terms of total effect, Lund Kennedy score of nasal endoscope and Lund Mackay score of sinus CT. For total effect, the curative effects of external herbal medicine and external herbal medicine + herbal medicine orally are better than those of nasal saline, external herbal medicine + herbal medicine orally and nasal saline + herbal medicine orally are better than those of nasal saline + Western medicine orally. The differences in the remaining comparisons are not statistically significant. For the Lund Kennedy score, the curative effects of nasal saline + herbal medicine orally were better than those of nasal saline. For the Lund Mackay score, the results of direct and indirect comparisons between groups were not statistically significant. The top three efficacy ranking results for total effect were external herbal medicine + herbal medicine orally, nasal saline + herbal medicine orally, and Herbal medicine orally. For Lund Kennedy score, the top three efficacy ranking results were nasal saline + herbal medicine orally, herbal medicine orally, external herbal medicine, while Herbal medicine orally, nasal saline + western medicine orally, nasal saline + herbal medicine orally for Lund Mackay score. The results above indicated that oral or external herbal medicine has better efficacy in the treatment of radiation sinusitis after radiotherapy for NPC. There was only one involved article [16] about topical application of herbal medicine on the skin. We can conclude that the combination of saline or herbal medicine through nasal cavities and herbal medicine taken orally has certain advantages, probably because the nasal mucosal cilia transport function cannot be restored in a short period of time, and pus, pus snot, necrotic material, exudate, etc. need external force to be thoroughly cleaned, so external applications such as nasal rinsing, negative pressure sinus replacement, and ultrasonic nebulized inhalation are particularly significant [20].

### Limitations

➀ The included studies were primarily small-sample-size studies, and the original data of the Lund Kennedy score and Lund Mackay score have substantial and unknown heterogeneity, which may damage the research statistically.
➁ Interventions of nasal saline, none and nasal saline + western medicine orally were only used as control groups in the included studies. As the medium of comparison in the remaining five interventions (external herbal medicine, herbal medicine orally, external herbal medicine + nasal saline, external herbal medicine + herbal medicine orally, nasal saline + herbal medicine orally), the research did not conduct a wide range of searches aimed at the three interventions. Therefore, the curative effect rankings among the three were not of reference value;
➂ The research categorized application methods (nasal rinsing, negative pressure sinus replacement, ultrasonic nebulized inhalation) into nasal methods macroscopically. The lack of detailed classification of those applications through nasal cavities may affect the evaluation of the efficacy of specific interventions. Subsequent studies are needed to classify and conduct more precise efficacy analysis.
➃ Only two of the included studies reported adverse reactions, which cannot entirely reflect safety.

## Conclusions

In conclusion, herbal medicine taken orally combined with herbal medicine or saline through nasal cavities have better efficacy in treating radiation sinusitis after radiotherapy for NPC than Nasal saline only. The included studies mostly used herbal medicine of nourishing yin and clearing heat, decongesting the nasal passage, antitumor and tonifying Qi and blood, such as Radix Glehniae, Herba Menthae, Radix Scutellaria, Radix Gentiana, Flos Chrysanthemi Indici, Spina Gleditsiae; Fructus Xanthii, Radix Angelicae Dahuricae, Flos Magnoliae; Hedyotis Diffusa, Herba Scutellariae Barbatae; Radix Astragali, Rhizoma ChuanXiong, etc.. Oral and nasal herbal medicine combined with nasal saline have a significant improvement in efficacy in terms of total effect and Lund Kennedy score. However, due to the small sample size of recent clinical studies and the macroscopic classification of this study, more thorough and high-quality studies are required for verification.

### Supplementary Information

Below is the link to the electronic supplementary material.

Supplementary file1 (DOCX 1140 KB)

## Data Availability

All data analyzed during this study are included in this published article. The details of the systematic review registration are available in PROSPERO (https://www.crd.york.ac.uk/prospero/).

## Abbreviations

CI: Credibility interval
CNKI: China national knowledge infrastructure
CQVIP: Chongqing VIP database
CSCO: Chinese Society of Clinical Oncology
IF: Inconsistency factors
IMRT: Intensity modulated radiotherapy
MD: Mean difference
NCCN: National Comprehensive Cancer Network
NMA: Network meta-analysis
NPC: Nasopharyngeal carcinoma
OR: Odds ratio
PICOS: P-population; I-intervention; C-comparison; O-outcome; S-study design
RCTs: Randomized controlled trials
RoB 2: Risk of bias 2
SD: Standard deviation
SE: Standard error
SUCRA: Surface under the cumulative ranking curve
TCM: Traditional Chinese Medicine
