# Supplementary material for: Traditional Chinese medicine treatment in sinusitis after radiotherapy for nasopharyngeal carcinoma: a systematic review and network meta-analysis
Source: Eur Arch Otorhinolaryngol. 2023 Sep 14;281(1):1–11. doi: 10.1007/s00405-023-08221-4 (PMC10764406; doi:10.1007/s00405-023-08221-4)
Supplement: Supplementary file 1 — Supplementary file1 (DOCX 1140 KB) [file 405_2023_8221_MOESM1_ESM.docx]

**Supplemental Table 1 PubMed search strategy**

| **#1** | Nasopharyngeal Carcinoma[MeSH] OR Nose Neoplasms[MeSH] |
| --- | --- |
| **#2** | Nasopharyngeal Cancer[Tiab] OR Nasopharyngeal Neoplasms[Tiab] OR Carcinoma, Nasopharyngeal[Tiab] OR Cancer of Nasopharynx[Tiab] OR Nasopharynx Cancers[Tiab] OR Nasopharyngeal malignant neoplasm[Tiab] OR Nasopharyngeal malignancy[Tiab] OR nose tumor[Tiab] OR Nasal Neoplasms[Tiab] OR Nose Cancer[Tiab] |
| **#3** | #1 OR #2 |
| **#4** | Radiotherapy[MeSH] OR Radiosurgery[MeSH] |
| **#5** | Radiation Therapy[Tiab] OR Targeted Radiotherapy[Tiab] OR Radiation Treatment[Tiab] OR X-ray therapy[Tiab] OR radioisotope therapy[Tiab] OR Stereotactic Radiation[Tiab] OR Stereotactic Radiosurgery[Tiab] OR intensity-modulated radiotherapy[Tiab] |
| **#6** | #4 OR #5 |
| **#7** | Sinusitis[MeSH] OR Paranasal Sinus Diseases [MeSH] |
| **#8** | Complications[Tiab] OR Sequelae[Tiab] OR sequels[Tiab] OR associated disease[Tiab] OR nose disease[Tiab] OR Nasal Obstruction[Tiab] OR Paranasal Sinus Diseases[Tiab] OR Sinus Infection[Tiab] |
| **#9** | #7 OR #8 |
| **#10** | Traditional Chinese Medicine[MeSH] OR Herbal Medicine[MeSH]OR Acupuncture[MeSH] OR Acupuncture therapy[MeSH] |
| **#11** | Herbalism[Tiab] OR Chinese Herbal Drugs[Tiab] OR Chinese Traditional Medicine[Tiab] OR TCM[Tiab] OR Chinese herbs[Tiab] OR Chinese medicine[Tiab] OR Chinese therapy[Tiab]* |
| **#12** | #10 OR #11 |
| **#13** | #3 AND #6 AND #9 AND #12 |

**Supplemental Table 2 Baseline characteristics included in the network meta-analysis.**

| **Study ID** | **Group** | | **Age**  **(mean±SD)** | **Staging** | **Intervention** | | **Outcome** |
| --- | --- | --- | --- | --- | --- | --- | --- |
|  | **exp** | **com** |  |  | **exp** | **com** |  |
| Li Y 2016 | 56 | 56 | 51.0±7.2/52.0±6.6 | / | A | B | ① |
| Ye FY 2020^[14]^ | 58 | 58 | 46.78±8.39/47.57±7.84 | Ⅰ-40;Ⅱ-16; Ⅲ-2 例/Ⅰ-45; Ⅱ-11; Ⅲ-2 | G | B | ① |
| Shi H 2012^[15]^ | 30 | 30 | 55.5±18.4/56.8±20.6 | / | H | D | ① |
| Zhang WJ 2011^[16]^ | 30 | 30 | 42 | / | I | B | ① |
| Peng J 2010^[17]^ | 27 | 27 | 18-58/21-62 | / | F | C | ① |
| Qu T 2017^[18]^ | 45 | 45 | 51.2±1.3/50.1±1.2 | Ⅰ-7; Ⅱ21; Ⅲ-17/Ⅰ-8; Ⅱ-19; Ⅲ-18 | D | B | ①②③ |
| Li YW 2015^[19]^ | 52 | 50 | 57.7±2.9/57.4±3.1 | Ⅰ-3; Ⅱ-27; Ⅲ-18; Ⅳ-4/Ⅰ-2; Ⅱ-25; Ⅲ-19; Ⅳ-4 | I | B | ①②③ |
| Qiao GY 2014^[20]^ | 35 | 35 | 56±12/58±10 | / | I | B | ②③ |
| Huang XL 2009^[21]^ | 63 | 63 | 48.6 | / | A | B | ① |
| Qiao GY 2019^[22]^ | 40 | 40 | 56±12/58±10 | Ⅰ-3; Ⅱ-18; Ⅲ-17; Ⅳ-2/Ⅰ-2; Ⅱ-19; Ⅲ-14; Ⅳ-3 | A | B | ②③ |
| Mao FM 2006^[23]^ | 35 | 33 | 42 | Ⅱ-28; Ⅲ-31; Ⅳ-9 | A | B | ① |
| Guo S 2019 | 15 | 15 | 49.07±12.79/47.07±10.22 | / | A | B | ①② |
| Yan W 2019^[24]^ | 40 | 40 | 64.82±5.83/64.26±5.63 | Ⅰ-4; Ⅱ-25; Ⅲ-11/Ⅰ-3; Ⅱ-25; Ⅲ-12 | A | C | ①② |
| Nie CY 2016^[25]^ | 70 | 70 | 58.6±6.3/57.2±6.9 | Ⅰ-12; Ⅱ-20; Ⅲ-28; Ⅳ-10/Ⅰ-14; Ⅱ-27; Ⅲ-23; Ⅳ-6 | I | J | ①③ |
| Liao SH 2007^[26]^ | 60 | 62 | 45 | Ⅰ-12; Ⅱ-50; Ⅲ-36; Ⅳ-24 | A | B |  |

(A-External herbal medicine, B-Nasal saline, C-None, D-Herbal medicine orally, G-External herbal medicine+ Nasal saline, H-External herbal medicine+ Herbal medicine orally, I-Nasal saline+ Herbal medicine orally, J-Nasal saline+ Western medicine orally)


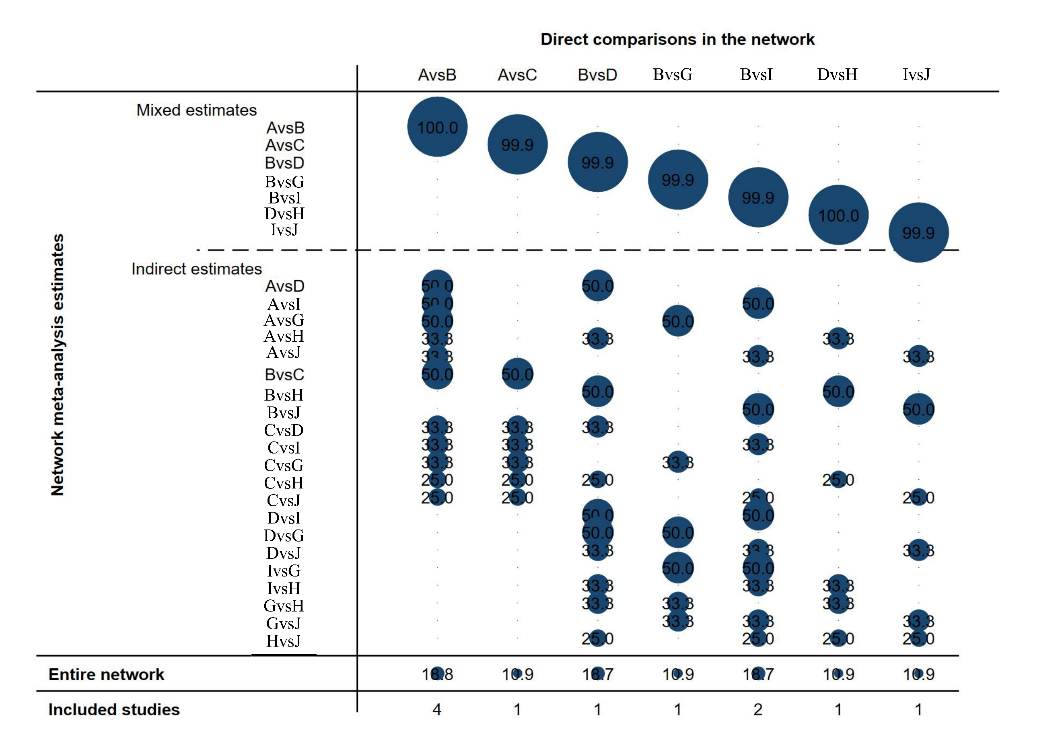


**Supplemental Figure 1 Contribution plots for treatments for total effect.**

(A-External herbal medicine, B-Nasal saline, C-None, D-Herbal medicine orally, G-External herbal medicine+ Nasal saline, H-External herbal medicine+ Herbal medicine orally, I-Nasal saline+ Herbal medicine orally, J-Nasal saline+ Western medicine orally) The size of each circle is proportional to the weight attached to each direct or indirect summary effect, and the numbers express the weights as percentages.


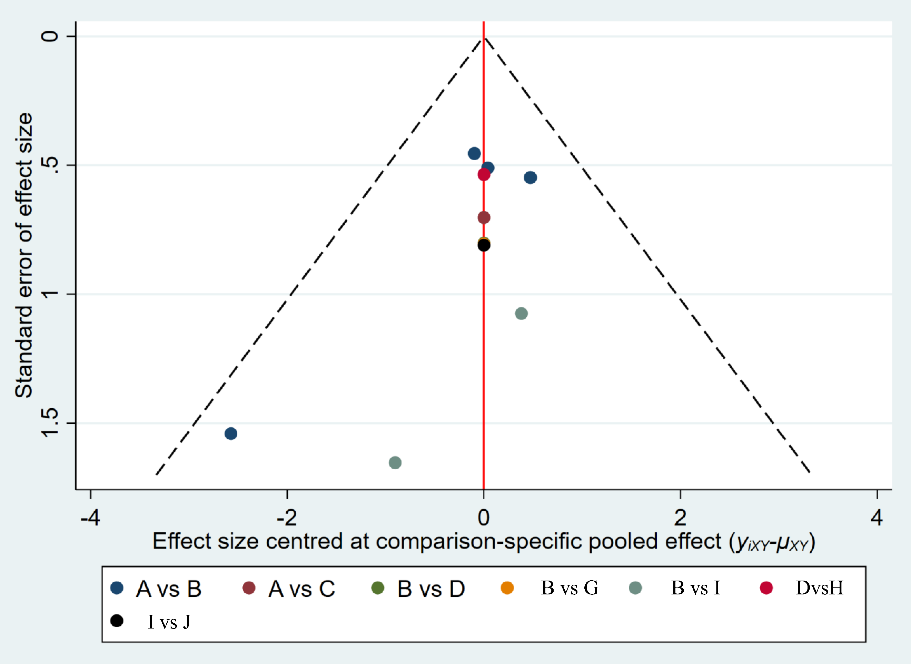


**Supplemental Figure 2 Funnel plot assessment of publication bias for total effect**

**（**A-External herbal medicine, B-Nasal saline, C-None, D-Herbal medicine orally, G-External herbal medicine+ Nasal saline, H-External herbal medicine+ Herbal medicine orally, I-Nasal saline+ Herbal medicine orally, J-Nasal saline+ Western medicine orally**）**Most dots are symmetrically distributed on both sides of the vertical line of the X=0 at the top of the funnel diagram, indicating a low possibility of both publication bias and small sample effect.


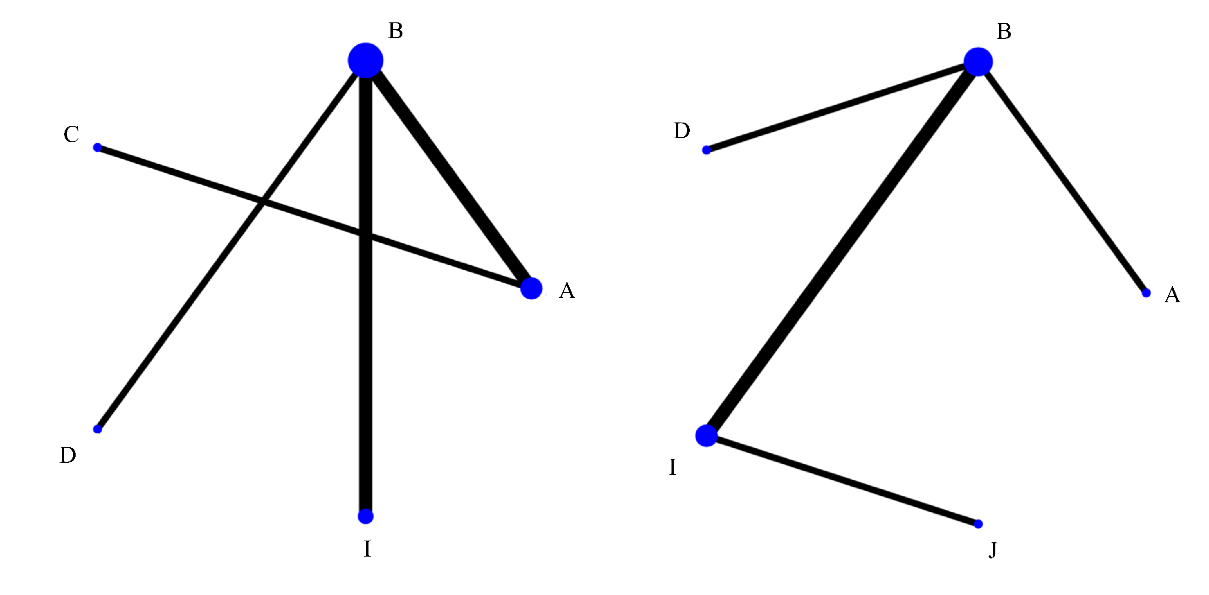


**Supplemental Figure 3 Network diagram comparing treatment outcomes for Lund Kennedy and Lund Mackay.**

(A-External herbal medicine, B-Nasal saline, C-None, D-Herbal medicine orally, I-Nasal saline+ Herbal medicine orally, J-Nasal saline+ Western medicine orally) The diameter of each dot represents the proportional total weight of all trials in the network that investigated that intervention, while the thickness of each line connecting 2 interventions is proportional to the number of trials that investigated that pair of interventions.


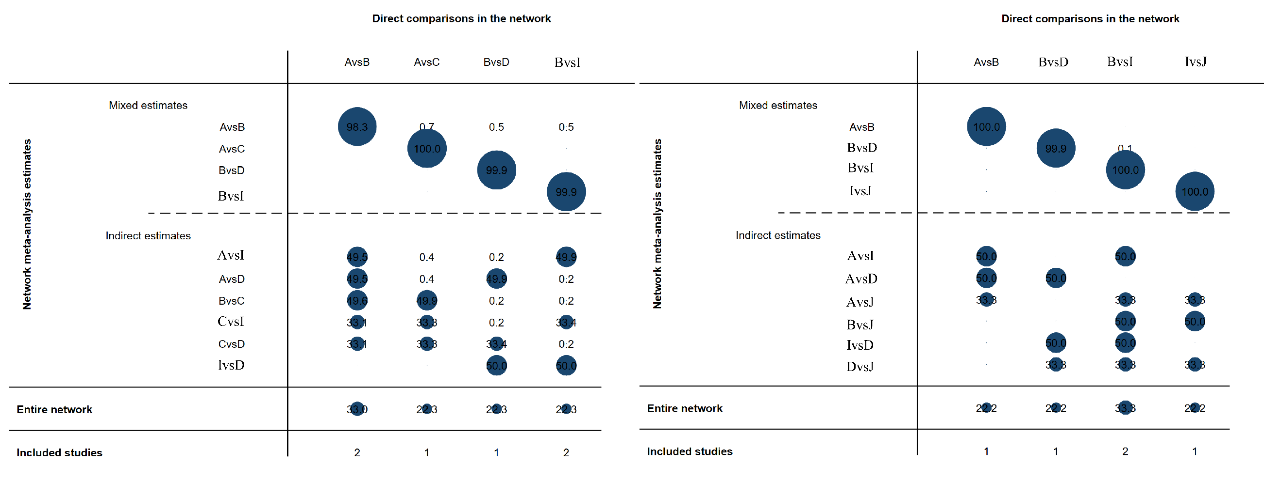


**Supplemental Figure 4 Contribution plots for treatments for Lund Kennedy and Lund Mackay.**

(A-External herbal medicine, B-Nasal saline, C-None, D-Herbal medicine orally, I-Nasal saline+ Herbal medicine orally, J-Nasal saline+ Western medicine orally) The size of each circle is proportional to the weight attached to each direct or indirect summary effect, and the numbers express the weights as percentages.


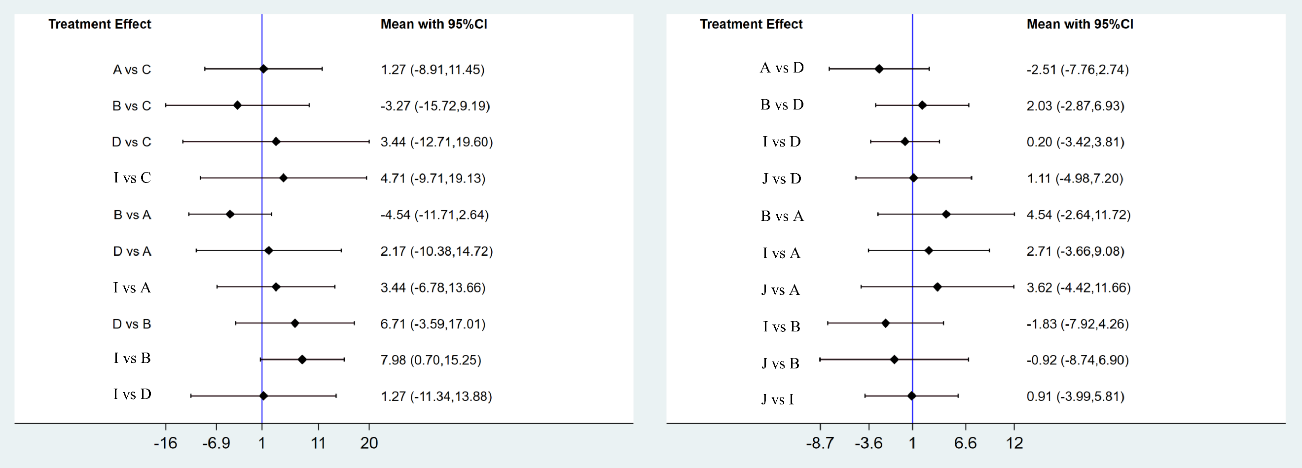


**Supplemental Figure 5 Forest plot of treatment differences for Lund Kennedy and Lund Mackay.**

(A-External herbal medicine, B-Nasal saline, C-None, D-Herbal medicine orally, I-Nasal saline+ Herbal medicine orally, J-Nasal saline+ Western medicine orally) The ineffectiveness line (vertical line, X=1) indicates an equal ratio. Each horizontal line connects the upper and lower limits of the 95% confidence interval for the study, and the length of the lines indicates the range of the confidence interval. If the line crossed=1, the study was not statistically significant. If the line totally falls on the left side of X=1, it indicates worse efficacy, and the right side indicates the opposite. The diamond-shaped blocks are locations corresponding to the OR values.

**Supplemental Table 3 Inverted triangle diagram for Lund Kennedy.**

| A | 0.01 (0.00,13.99) | 8.78 (0.00,2.48e+06) | 31.19 (0.00,854768.57) | 0.28 (0.00,7400.95) |
| --- | --- | --- | --- | --- |
| 93.50 (0.07,122278.43) | B | 820.92 (0.03,2.43e+07) | 2915.79 (2.02,4.21e+06) | 26.21 (0.00,6.70e+06) |
| 0.11 (0.00,32129.45) | 0.00 (0.00,36.10) | D | 3.55 (0.00,1.06e+06) | 0.03 (0.00,331632.94) |
| 0.03 (0.00,878.88) | 0.00 (0.00,0.50) | 0.28 (0.00,84168.69) | I | 0.01 (0.00,16452.74) |
| 3.57 (0.00,94210.01) | 0.04 (0.00,9756.92) | 31.33 (0.00,3.25e+08) | 111.27 (0.00,2.04e+08) | C |

The yellow table cells represent interventions. **(**A-External herbal medicine, B-Nasal saline, C-None, D-Herbal medicine orally, I-Nasal saline+ Herbal medicine orally, J-Nasal saline+ Western medicine orally**)** The green table cells represent the combined effect size, which can be used to compare the curative effect between the interventions in the column and the line.

**
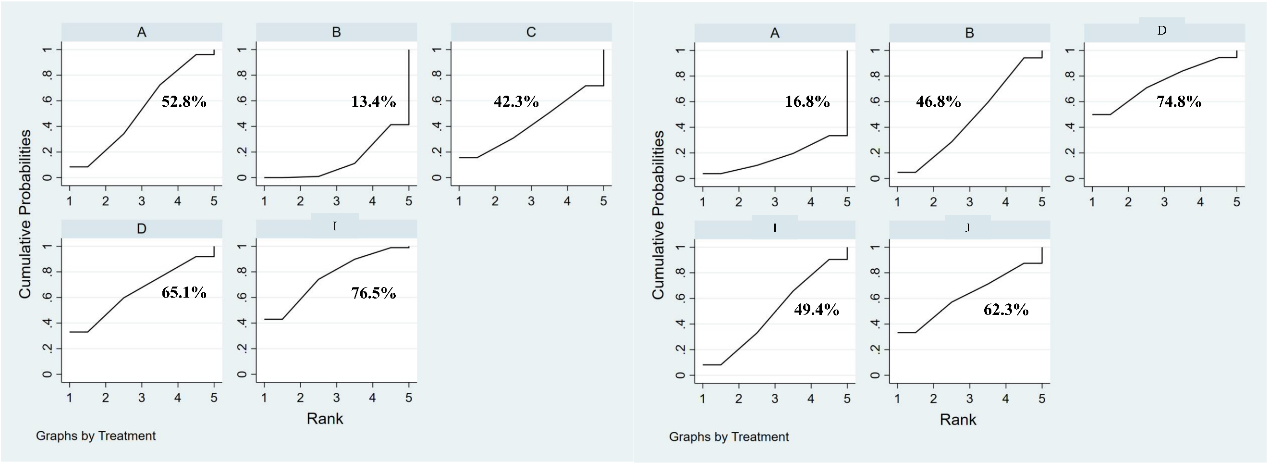
**

**Supplemental Figure 6 Surface under the cumulative ranking curves for Lund Kennedy and Lund Mackay.**

(A-External herbal medicine, B-Nasal saline, C-None, D-Herbal medicine orally, I-Nasal saline+ Herbal medicine orally, J-Nasal saline+ Western medicine orally) The Y-axis represents cumulative probability, and the X-axis represents rank. When comparing the cumulative probability of the same control rank, the higher ranking (5→1) with a higher cumulative probability means a better curative effect.


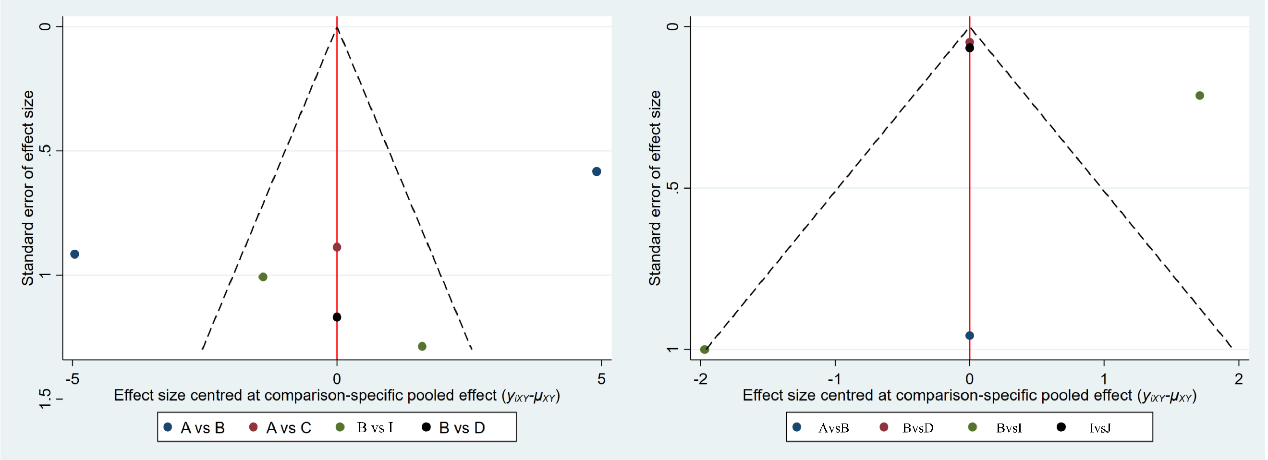


**Supplemental Figure 7 Funnel plot assessment of publication bias for Lund Kennedy and Lund Mackay.**

**（**A-External herbal medicine, B-Nasal saline, C-None, D-Herbal medicine orally, I-Nasal saline+ Herbal medicine orally, J-Nasal saline+ Western medicine orally**）**Few dots are symmetrically distributed on both sides of the vertical line of the X=0 at the top of the funnel diagram, indicating possibility of both publication bias and small sample effect.

**Supplemental Table 4 Inverted triangle diagram for Lund Mackay.**

| A | 93.69 (0.07,122780.21) | 15.03 (0.03,8782.43) | 37.33 (0.01,115524.65) | 12.30 (0.06,2334.89) |
| --- | --- | --- | --- | --- |
| 0.01 (0.00,13.99) | D | 0.16 (0.00,70.73) | 0.40 (0.00,988.19) | 0.13 (0.00,17.63) |
| 0.07 (0.00,38.90) | 6.24 (0.01,2749.83) | I | 2.48 (0.02,333.80) | 0.82 (0.02,30.43) |
| 0.03 (0.00,82.90) | 2.51 (0.00,6224.99) | 0.40 (0.00,54.09) | J | 0.33 (0.00,145.45) |
| 0.08 (0.00,15.42) | 7.61 (0.06,1022.31) | 1.22 (0.03,45.37) | 3.03 (0.01,1338.59) | B |

The yellow table cells represent interventions. **(**A-External herbal medicine, B-Nasal saline, D-Herbal medicine orally, I-Nasal saline+ Herbal medicine orally, J-Nasal saline+ Western medicine orally**)** The results showed no statistical significance.
